# Similarities and Differences in Associations between Duration of Untreated Psychosis (DUP) and Demographic, Premorbid, and Symptom Severity Measures in Two Samples of First-Episode Psychosis Patients from Mexico and the United States

**DOI:** 10.1007/s11126-020-09736-3

**Published:** 2020-09

**Authors:** Ana Fresan, Rogelio Apiquian, Rebeca Robles-García, Carlos-Alfonso Tovilla Zarate, Pierfrancesco Maria Balducci, Beth Broussard, Claire Ramsay Wan, Michael T. Compton

**Affiliations:** Subdirección de Investigaciones Clínicas, Instituto Nacional de Psiquiatría Ramón de la Fuente Muñíz, Mexico City, D.F., Mexico; División de Ciencias del Comportamiento y del Desarrollo, Universidad de las Américas A.C., Mexico City, D.F., Mexico; Dirección de Investigaciones Epidemiológicas y Sociales, Instituto Nacional de Psiquiatría Ramón de la Fuente Muñíz, Mexico City, DF., Mexico; División Académica Multidisciplinaria de Comalcalco, Universidad Juárez Autónoma de Tabasco, Tabasco, Mexico; Scuola di Specializzazione in Psichiatria, Dipartimento di Medicina, Università degli Studi di Perugia, Perugia, Italy; Emory University School of Medicine, Department of Psychiatry and Behavioral Sciences, Atlanta, GA, USA; Emory Healthcare, Atlanta, GA, USA; Columbia University College of Physicians & Surgeons, Department of Psychiatry, New York State Psychiatric Institute, New York, NY, USA

**Keywords:** Duration of untreated psychosis, First-episode psychosis, Psychosis, Schizophrenia

## Abstract

**Objective:**

Early-psychosis researchers have documented that duration of untreated psychosis (DUP) is an important predictor of outcomes in first-episode psychosis. Very few cross-national studies have been conducted, and none have been carried out involving patients from both Mexico and the U.S. We collaborated to answer three questions: (1)Are DUP estimates similar in two very different settings and samples? (2)Are demographic variables, premorbid adjustment, and symptom severity similarly related to DUP in the two different settings? (3)Does the same set of variables account for a similar proportion of variance in DUP in the two settings?

**Methods:**

Data on sociodemographic characteristics, premorbid adjustment, symptom severity, and DUP were available for 145 Mexican and 247 U.S. first-episode psychosis patients. DUP was compared, and bivariate analyses and multiple linear regressions were carried out in each sample.

**Results:**

DUP estimates were similar (medians of 35 weeks in Mexico and 38 weeks in the U.S.). In the Mexican sample, DUP was associated with gender, employment status, premorbid social adjustment, and positive symptom severity (explaining 18% of variance). In the U.S. sample, DUP was associated with age, employment status, premorbid social adjustment, and positive symptom severity (but in the opposite direction of that observed in the Mexican sample), accounting for 25% of variance.

**Conclusions:**

Additional cross-national collaborations examining key facets of early-course psychotic disorders, including DUP, will clarify the extent of generalizability of findings, strengthen partnerships for more internationally relevant studies, and support the global movement to help young people struggling with first-episode psychosis and their families.

## Introduction

Studies on first-episode psychosis from around the world have consistently shown duration of untreated psychosis (DUP)—often defined as the time interval from onset of frank psychotic symptoms (more specifically, the presence of delusions and hallucinations) to the first contact with a psychiatric facility to receive adequate pharmacological treatment—to be an important predictor of clinical and social outcomes in patients with first-episode psychosis [1]. A longer DUP in adult first-episode patients has been related to greater symptom severity at initial treatment, poorer response to antipsychotic medication, and diminished quality of life [2–6].

The specific mechanisms underlying the association between DUP and outcome variables are not yet clearly identified. It is still unknown if DUP *causes* poor outcomes or if individuals at risk for poor outcome receive specialized treatment long after the onset of symptoms [7]. It is clear, however, that DUP temporally precedes measures of psychotic features of the disorder at initial specialized treatment contact, that it is likely modifiable [3], and that it is one of the main variables targeted by early intervention services [8–10].

Although longer DUP can be considered a risk factor for poorer outcomes in patients with psychosis, little is known about the determinants of a prolonged DUP [7]. Some studies have indicated that factors associated with longer DUP include male gender, unemployment, being single, lack of family support, stigma, and behaviors related to social isolation [11–15]. Some researchers have suggested that premorbid adjustment (also referred to as premorbid functioning) may be an important variable related to DUP [16–18], and it even has been considered a moderator of the association between DUP and symptomatology [3, 19].

Lower premorbid adjustment is reflected by a poorer adaptation to school, lower academic performance, and limited social relationships during childhood and adolescence. If an insidious illness onset begins during this time, early manifestations of the disorder, such as predominant negative symptoms, are often misattributed to other circumstances rather than a serious mental illness, such as substance use, difficulties at school, or simply behaviors considered to be characteristic of adolescence [11, 13, 20–22]. As life becomes more challenging during emotional development and more skills are needed in adolescence than in childhood, with this gradual onset of negative or psychotic symptoms, both patients and their relatives may have habituated and even made lifestyle adjustments, resulting in a longer DUP [9]. Therefore, the associations between impaired premorbid adjustment, a longer DUP, and clinical outcomes may be impacted by other factors, not to mention cultural background and healthcare accessibility [15, 17].

In this study, we leveraged a collaboration between Mexican and U.S. early psychosis researchers to study two samples with regard to key variables pertaining to first-episode psychosis. We specifically sought to answer three research questions about DUP. First, are DUP estimates similar or different in the two very different settings and samples? Second, are basic demographic variables, premorbid adjustment scores, and symptom severity scores similarly related to DUP in the two settings and samples? Third, does the same set of variables account for a similar portion of variance in DUP in the two different settings and samples?

## Methods

### Settings and Samples

Patients in Mexico were consecutively recruited from both the inpatient and outpatient services of the Instituto Nacional de Psiquiatría Ramón de la Fuente Muñíz (INPRFM), a highly specialized mental health center in Mexico City, dedicated to research, education, and treatment of psychiatric patients. A total of 145 patients, enrolled from the prospective Mexican First-Episode Psychotic Study [23] were included in the present analysis. All patients were experiencing their first episode of psychosis, defined as the first contact in life with a specialized service of psychiatry due to psychotic symptoms [24] and had never received any antipsychotic treatment and had not been previously hospitalized. Patients were excluded if they had concomitant medical or neurological illness, had current substance abuse or a history of substance dependence, had a history of bipolar disorder, or were too agitated to participate in the clinical research interview. The study was approved by the ethics review board of the INPRFM and written informed consent was obtained after the procedures had been fully explained to patients and their relatives. All information was collected by a trained, doctoral-level psychiatrist who conducted the clinical interview with patients and their relatives.

With regard to the settings from which the U.S. participant were drawn, as part of a project examining the effects of premorbid marijuana use on early-course psychosis [25], consecutively admitted first-episode patients were approached about potential participation in a cross-sectional/retrospective study. First-episode psychosis was operationalized as having received <3 months of prior antipsychotic treatment and having never been hospitalized for psychosis earlier than three months prior to index admission (though the vast majority were completely naïve to any psychiatric treatment prior to admission). A total of 247 participants—213 (86.2%) of whom were African American—were enrolled from August 2008 to June 2013 from three inpatient psychiatric units in Atlanta, Georgia (n=225) and three units in Washington, D.C. (n=22). Eligible patients were 18–40 years of age, English-speaking, and able to give informed consent. Exclusion criteria included known or suspected mental retardation, a Mini-Mental State Examination [26, 27] score of <24, or the presence of a major medical condition compromising ability to participate. Once psychotic symptoms were stabilized sufficiently for informed consent and participation, trained masters- or doctoral-level assessors conducted the in-depth assessments for the parent study.

At both sites, after a clinical interview with the patient and his or her relatives, a trained clinical researcher made a diagnosis using the *Structured Clinical Interview for DSM-IV Axis I Disorders* (SCID-I) [28], to confirm the presence of a primary, non-affective psychotic disorder.

### Measures and Rating Scales

The *Premorbid Adjustment Scale* (PAS) [29] assesses the degree to which a person has successfully attained certain developmental goals at various life stages preceding the initial onset of psychotic symptoms. The instrument has been used widely in schizophrenia research, and reliability, validity, and predictive utility have been reported previously [29–32]. Functioning is assessed across four age periods: *childhood* (≤11 years), *early adolescence* (12–15 years), *late adolescence* (16–18 years), and *adulthood* (≥19 years). Functioning in each of these age periods is assessed across five major psychosocial domains that are rated from 0 (normal adjustment) to6 (severe impairment): *sociability and withdrawal*, *peer relationships*, *scholastic performance*, *adaptation to school*, and *social-sexual functioning*. Social-sexual functioning is not included as a psychosocial domain during the childhood period, while scholastic performance and adaptation to school are not measured during the adulthood period. The adulthood period was not assessed in the present study. Thus, in childhood, academic functioning includes *scholastic performance* and *adaptation to school*, and the social functioning encompasses *sociability and withdrawal* and *peer relationships*. In both early adolescence and late adolescence, academic functioning is comprised of *scholastic performance* and *adaptation to school*, and social functioning includes *sociability and withdrawal*, *peer relationships*, and *social-sexual functioning*. At both the Mexican and U.S. sites, functioning was not rated in a respective age period if prodromal or psychotic symptoms began during or within one year of that period, as described previously [33].

Positive and negative symptom severity was assessed at both sites with the commonly used *Positive and Negative Syndrome Scale* (PANSS) [34]. The PANSS includes 30 symptoms, measured at present and over the past month on a scale from 1=absent to 7=extreme. The original PANSS positive, negative, and general psychopathology symptom subscales were used. The intra-class correlation (ICC) coefficients for inter-rater reliability were between 0.80 and 0.91 for the different rating scales at the Mexican site [35]. At the U.S. site, ICCs for inter-rater reliability of the PANSS subscale scores were: 0.92 for the positive symptom subscale, 0.92 for the negative symptom subscale, and 0.67 for the general psychopathology subscale.

At the Mexican site, DUP was measured following the criteria proposed by Larsen [36] and was defined as the time between first onset of psychotic symptoms and first contact with a psychiatric service for receiving specific antipsychotic treatment for symptoms. Symptom onset was defined as the presence of delusions, conceptual disorganization, hallucinations, grandiosity, suspiciousness, or unusual thought content, rated as ≥3 according to PANSS items P1, P2, P3, P5, P6 and G9; this information was obtained by a retrospective evaluation during the in-depth initial interview with the patient and his or her family.

At the U.S. site, age at onset of psychosis and DUP (duration in weeks from onset of hallucinations or delusions, whichever came first, to first hospital admission) were determined using the *Symptom Onset in Schizophrenia* (SOS) inventory [37]. The SOS criteria were used to determine when hallucinations and/or delusions met the threshold for psychosis. Using all available information, a consensus-based best-estimate date identified the onset of symptoms; specifically, when the severity of the symptom met clinical criteria, and the symptom occurred often enough to meet or exceed the required frequency. Prior reports describe the U.S. research team’s standardized approach to using the SOS and deriving age at onset and DUP using consensus-based best-estimate methods [11, 38–40].

### Data Analyses

Bivariate analyses for comparisons between the Mexican and U.S. samples of first-episode psychosis patients were carried out using χ^2^ tests of association for categorical variables, independent samples Student’s *t* tests for continuous variables with approximately normal distributions (based on examination of descriptive statistics and the Kolmogorov-Smirnov test), and Mann-Whitney *U* tests for continuous variables that were not normally distributed. Correlations were computed using Pearson and Spearman correlation coefficients as appropriate.

To assess the independent effects of correlates of DUP in both samples, we computed a log transformation of DUP, ln(DUP+1), which normalized the distribution to allow for multiple linear regression models.

## Results

### Comparisons of Sociodemographic and Clinical Characteristics of the Two Samples

We first compared our two first-episode psychosis samples in terms of basic demographic variables. As shown in Table 1, the two samples differed substantially. Specifically, patients in the Mexican sample were older (median age of 27.0 compared to 23.0; *z*=3.99, *p*<0.001) and more likely to be unemployed (at a trend level). Patients in the U.S. sample had completed more years of education (median of 12.0 compared to 11.0; *z*=3.67, *p*<0.001), were more likely to be male (74.5% compared to 58.6%; χ^2^=10.69, *df*=1, *p*=0.001), and were more likely to be single (94.7% compared to 78.6%; χ^2^=23.82, *df*=1, *p*<0.001).

**Figure 1.**
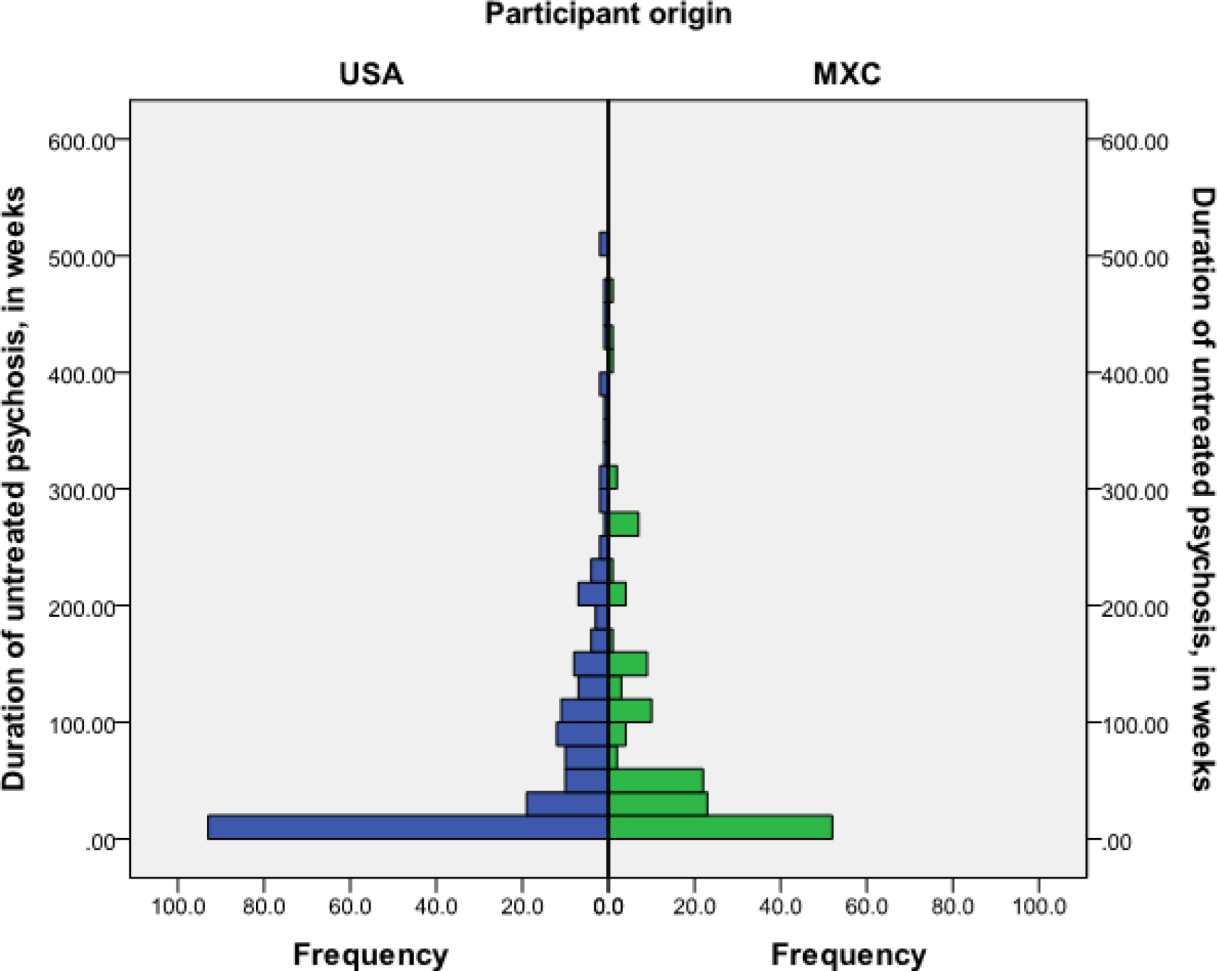
Distributions of DUP in Weeks in the U.S. and Mexican Samples of First-Episode Psychosis Patients† **†** To improve visual quality of the histograms, 19 values with a DUP of >10 years were excluded from the figure.

In terms of PAS scores, patients in the Mexican sample had lower PAS academic adjustment scores (indicating better premorbid academic adjustment) across all three age groups. However, PAS social adjustment scores did not differ. Also shown in Table 1, duration of untreated psychosis did not differ between the Mexican and U.S. samples (medians of 35.0 and 38.0; *z*=0.02, *p*=0.99); the distributions of DUP are shown in Figure 1. Symptom severity, based on the PANSS positive symptom subscale, negative symptom subscale, and general psychopathology symptom subscale did not differ significantly across the two samples.

### Inter-Correlations among Premorbid Adjustment and Symptom Scores in the Two Samples

Inter-correlations among PAS subscale scores, in both samples, are shown in Table 2. As expected, correlations between the three academic adjustment scores were statistically significant (all *p*<0.01) and in the moderate range (the average of the three correlations was 0.46 in the Mexican sample and 0.49 in the U.S. sample). Correlations between the three social adjustment scores were also statistically significant (all *p*<0.01) and larger (the average of the three correlations was 0.68 in the Mexican sample and 0.58 in the U.S. sample). On the other hand, correlations between the three academic adjustment scores and the three social adjustment scores were only modest (the average of the nine correlations was 0.21 in the Mexican sample (range, 0.05–0.38) and 0.17 in the U.S. sample (range, 0.08–0.34)).

With regard to inter-correlations among the three PANSS symptom severity scores, findings were again consistent in the Mexican and U.S. samples of first-episode patients (Table 3). Specifically, positive symptom and negative symptom subscale scores were least correlated (*r*=0.15 in Mexican patients and *r*=0.23 in U.S. patients), and negative symptom and general psychopathology symptom subscale scores were most correlated (*r*=0.55 and *r*=0.59, respectively).

### Correlates of Duration of Untreated Psychosis in the Two Samples

In terms of associations between DUP and the five demographic variables (age, years of education, gender, marital status (single versus married or living with a partner), and employment status), in the U.S. sample, age and employment status were associated with DUP. Age was directly correlated with DUP (ρ=0.46, *p*<0.001), though it was not associated with DUP in the Mexican sample (ρ=0.09, *p*=0.27). Also in the U.S. sample, employment status was associated with DUP at a trend level (median DUP of 25.0 weeks among the 71 with paid employment and 62.5 weeks among the 152 who were unemployed; *z*=1.85, *p*=0.065). In the Mexican sample, both gender (median DUP of 25.0 among the 60 females and 52.0 among the 85 males; *z*=2.26, *p*=0.025) and employment status were associated with DUP (median DUP of 17.0 among the 34 with paid employment and 52.0 among the 111 who were unemployed; *z*=2.64, *p*=0.008).

Correlations among DUP, PAS academic adjustment (an average score across the three age periods), PAS social adjustment (again, an average across the three age periods), and PANSS scores are shown in Table 4. In the Mexican and U.S. samples, poorer PAS academic adjustment (i.e., a higher score) was minimally associated with a longer DUP (ρ=0.14 and ρ=0.12), and poorer PAS social adjustment was modestly associated with a longer DUP (ρ=0.25 and ρ=0.20, both *p*<0.01). In both samples, DUP was associated neither with negative symptom severity nor general psychopathology symptom severity. In the Mexican sample, longer DUP was associated with a lesser severity of positive symptoms (ρ=−0.23, *p*<0.05), but in the U.S. sample, longer DUP was associated with a greater severity of positive symptoms (ρ=0.17, *p*<0.05).

### Regression Models Pertaining to Duration of Untreated Psychosis in the Two Samples

In each sample, multiple linear regressions were run, including variables found to be associated with DUP in the aforementioned bivariate tests. Results of these two models are shown in Table 5. In the model involving the Mexican sample, the four independent variables (gender, employment status, PAS social adjustment score, and PANSS positive symptom severity) accounted for 18% of the variance in DUP *(n*=111, *F*=5.70, *p*<0.001). Similarly, in the model pertaining to the U.S. sample, the four independent variables (age, employment status, PAS social adjustment score, and PANSS positive symptom severity) accounted for 25% of the variance in DUP (*n*=205, *F*=16.81, *p*<0.001).

## 4. Discussion

Several interesting findings emerged from this analysis, which represents one of the first cross-national comparisons of DUP and predictors/correlates of DUP, and the first to do so among first-episode samples in Mexico and the U.S. First, we found that the distributions of DUP—and the medians of DUP—were remarkably similar in these two very different settings and samples. Second, we observed similarities in variables associated with DUP; for example, premorbid *social* adjustment was related to DUP in both samples, though premorbid *academic* adjustment was not. Third, interestingly, positive symptom severity was associated with DUP in the two samples, but in contrasting ways. Fourth, we found that a similar set of four variables account for approximately one-fifth to one-quarter of the variance in DUP in the two different settings and samples.

With regard to the first of these findings, in our samples, the median DUP is around 8–9 months. This is consistent with findings in several other studies [6, 41]. One explanation for the somewhat surprising consistency is that emerging/evolving positive psychotic symptoms may often reach a “threshold of intolerability” at about 5–10 months, at which point families bring the person in for evaluation because they can no longer tolerate behavioral manifestations of symptoms, or they are finally confident that the problem is psychiatric in nature. Nevertheless, the DUP distributions in both samples analyzed in the present study indicate that some patients/families come in much sooner and others wait much longer. The latter could be related to stigma and lack of information about psychosis and its treatment. It has been demonstrated that stigma may represent a barrier that results in treatment delays [14, 42], and that early detection programs with public informational campaigns (which enhance community knowledge and reduce misconceptions) decrease DUP [43, 44].

Our similarities in correlates of DUP add more evidence regarding the importance of poor premorbid social adjustment in understanding DUP. This variable may produce longer DUP as poor social adjustment is related to decreased social support [45–47] (including friends who could notice symptoms and provide assistance for professional help-seeking). Previously, Drake and colleagues [48] found that longer DUP was predicted by worse social integration. The replicated association between poor premorbid social adjustment and DUP suggests that methods to reduce DUP in both middle- and high-income countries should consider this predictor.

Regarding the relationship between DUP and positive symptoms, as expected due to the difficulty for the patient/family to recognize/accept a psychiatric disorder that needs to be evaluated and treated when positive symptoms are less severe or not present, a longer DUP was associated with *lesser* severity of this kind of symptoms in the Mexican sample. However, a longer DUP was associated with *greater* positive symptom severity in the U.S. sample. This apparently contradictory finding could be explained by the exacerbation of stigma in the presence of positive symptoms [49] and consequent discrimination and isolation of patients in the U.S. sample. It has been suggested that reported outcome differences amongst patients from developed and developing countries could be related to higher levels of stigma in high-income countries [50]. Additionally, patients in the U.S. sample were more likely to be single than patients from Mexico. It is possible that stigma may more negatively impact those patients who live alone and have no close significant others that encourage and help them to seek evaluation and treatment. Decades ago, House and colleagues [46] pointed out the effects that limited social networks would have on health, and this could be a factor in the association between longer DUP and greater positive symptom severity among the U.S. participants (who were less likely to be married and more likely to be living alone). Although in the Mexican sample more than 70% also reported being single, patients arrived always with a relative, and in Mexico, unmarried people usually live with a nuclear or extended family member who give them support as needed. It is clear that there are important social determinants—such as social tolerance and support, stigma and discrimination, and related constructs—that need to be further addressed to understand the mechanisms through which demographic and clinical variables impact DUP.

In comparing DUP estimates, predictors of DUP, and proportion of DUP explained by similar sets of predictors, we knew *a priori* that our two settings and samples were quite different; we view the findings as particularly informative in part because of this lack of similarity. In both samples, four predictors accounted for 18–25% of the variance in DUP.

Several methodological limitations should be noted. First, this was a secondary analysis of two existing but similar datasets that were combined for the purposes of this collaborative analysis. As such, there were subtle differences in the exact measurement methods for key variables, including DUP. Second, as is true of any study of DUP, this is a difficult construct to measure as it inevitably relies on retrospective recall of patients and their family members. Third, and also related to the fact that this was a secondary analysis, there are a host of other variables that would have ideally been measured, and which could have explained more variance in DUP. Despite these limitations, our analysis reveals the value of cross-national collaborations in examining key facets of the early course of psychotic disorders, including DUP. Such studies will clarify the extent of broad generalizability of findings even in quite different samples; strengthen partnerships for more rigorous and internationally relevant studies; and support the now global movement to help young people struggling with early psychosis and their families, who must navigate complex systems of care while facing diverse social and psychological challenges.

## Figures and Tables

**Table 1.** Comparisons of Demographic and Clinical Characteristics in the Two Study Samples

	*Mexican Sample (n=145)*	*U.S. Sample (n=247)*	*Test statistic, df, p*
***Basic Demographic Variables***			
Age, in years^a^	27.0 (12.0)	23.0 (6.0)	*z*=3.99, p<0.001
Years of school completed^a^	11.0 (4.0)	12.0 (2.0)	*z*=3.67, *p*<0.001
Gender, male	85 (58.6%)	184 (74.5%)	χ^2^=10.69, *df*=1, *p*=0.001
Marital status, single	114 (78.6%)	234 (94.7%)	χ^2^=23.82, *df*=l, *p*<0.001
Employment status, unemployed	111 (76.6%)	168 (68.0%)	χ^2^=3.24, *df*=1,*p*=0.07
***Premorbid Adjustment Scale***^a,b^			
Childhood, academic	1.0 (1.5)	1.5 (1.5)	*z*=3.13,*p*=0.002
Early adolescence, academic	2.0 (2.0)	2.5 (2.0)	*z*=4.39, *p*<0.001
Late adolescence, academic	1.0 (2.3)	3.0 (2.5)	*z*=3.82, *p* <0.001
Childhood, social	1.5 (2.0)	1.0 (1.5)	*z*=1.39, *p* =0.17
Early adolescence, social	0.5 (1.7)	1.3 (1.3)	*z*=1.36, p =0.17
Late adolescence, social	1.3 (2.0)	1.3 (1.3)	*z*=0.93, *p* =0.35
***Duration of Untreated Psychosis***^a^	35.0 (93.0)	38.0 (151)	*z*=0.02,*p*=0.99
***Positive and Negative Syndrome Scale***			
Positive symptom severity	24.6±5.1	23.9±5.6	*t*=−1.13, *df*=356,*p*=0.26
Negative symptom severity	21.9±7.6	22.7±6.5	*t*=0.90, *df*=185.28,*p*=0.37^c^
General psychopathology severity	46.4±8.4	45.9±9.3	*t*=−044, *df*=233.48,*p*=0.66^c^

a Because the distributions of variables were not normally distributed, Mann-Whitney U tests were used, and median (and inter-quartile range) are shown.

b Lower scores indicate better premorbid adjustment.

c Degrees of freedom adjusted due to a significant Levene’s test for equality of variances.

**Table 2.** Inter-correlations among Premorbid Adjustment Scale (PAS) Subscale Scores†

	Ch-Acad	EA-Acad	LA-Acad	Ch-Soc	EA-Soc	LA-Soc
Ch-Acad	–	0.58**	0.34**	0.34**	0.23**	0.10
EA-Acad	0.52**	–	0.56**	0.20**	0.17*	0.10
LA-Acad	0.28**	0.58**	–	0.08	0.11	0.24**
Ch-Soc	0.20*	0.14	0.05	–	0.60**	0.47**
EA-Soc	0.18	0.21*	0.27**	0.72**	–	0.66**
LA-Soc	0.14	0.28**	0.38**	0.55**	0.77**	–

† Spearman correlation coefficients are shown because the distributions of PAS scores were not normally distributed. Correlations from Mexican patients in un-shaded cells, correlations from U.S. patients in shaded cells.

Ch=Child, EA=Early adolescence, LA=Late adolescence, Acad=Academic, Soc=Social

* p<0.05

** p<0.01

**Table 3.** Inter-correlations among Positive and Negative Syndrome Scale (PANSS) Subscale Scores†

	Positive	Negative	General
Positive	–	0.23**	0.55**
Negative	0.15	–	0.59**
General	0.38**	0.55**	–

† Correlations from Mexican patients in un-shaded cells, correlations from U.S. patients in shaded cells.

* p<0.05

** p<0.01

**Table 4.** Correlations among Duration of Untreated Psychosis, Premorbid Adjustment Scale (PAS) Subscale Scores, and Positive and Negative Syndrome Scale (PANSS) Scores†

	*Mexican Sample (n=145)*	*U.S. Sample (n=247)*
PAS-Academic	0.14	0.12
PAS-Social	0.25**	0.20**
Positive Symptoms	−0.23*	0.17*
Negative Symptoms	0.02	0.04
General Psychopathology Symptoms	0.00	0.05

† Spearman correlation coefficients are shown because the distributions of DUP were not normally distributed. Correlations from Mexican patients in un-shaded cells, correlations from U.S. patients in shaded cells.

* p<0.05

** p<0.01

**Table 5.** Multiple Linear Regression Models, ln(DUP+1) as the Dependent Variable

Variable	β	t	P
***Mexican Sample***			
Gender	0.182	2.03	0.05
Employment status	−0.158	−1.78	0.08
PAS Social Adjustment	0.169	1.88	0.06
PANSS Positive Symptoms	−.275	−3,2	0.002
*n*=111, *F*=5.70, *p*<0.001, *R*=0.42, *R*^2^=0.18			
***U.S. Sample***			
Age	0.426	6.90	<0.001
Employment status	−0.101	−1.61	0.11
PAS Social Adjustment	0.165	2.65	0.009
PANSS Positive Symptoms	0.108	1.72	0.09
*n*=205, *F*=16.81, *p*<0.001, *R*=0.50, *R*^2^=0.25			
